# New Species in the Old World: Europe as a Frontier in Biodiversity Exploration, a Test Bed for 21st Century Taxonomy

**DOI:** 10.1371/journal.pone.0036881

**Published:** 2012-05-23

**Authors:** Benoît Fontaine, Kees van Achterberg, Miguel Angel Alonso-Zarazaga, Rafael Araujo, Manfred Asche, Horst Aspöck, Ulrike Aspöck, Paolo Audisio, Berend Aukema, Nicolas Bailly, Maria Balsamo, Ruud A. Bank, Carlo Belfiore, Wieslaw Bogdanowicz, Geoffrey Boxshall, Daniel Burckhardt, Przemysław Chylarecki, Louis Deharveng, Alain Dubois, Henrik Enghoff, Romolo Fochetti, Colin Fontaine, Olivier Gargominy, Maria Soledad Gomez Lopez, Daniel Goujet, Mark S. Harvey, Klaus-Gerhard Heller, Peter van Helsdingen, Hannelore Hoch, Yde De Jong, Ole Karsholt, Wouter Los, Wojciech Magowski, Jos A. Massard, Sandra J. McInnes, Luis F. Mendes, Eberhard Mey, Verner Michelsen, Alessandro Minelli, Juan M. Nieto Nafrıa, Erik J. van Nieukerken, Thomas Pape, Willy De Prins, Marian Ramos, Claudia Ricci, Cees Roselaar, Emilia Rota, Hendrik Segers, Tarmo Timm, Jan van Tol, Philippe Bouchet

**Affiliations:** 1 UMR 7204, Muséum National d’Histoire Naturelle, Paris, France; 2 NCB Naturalis, Leiden, The Netherlands; 3 Depto. de Biodiversidad y Biologıa Evolutiva, Museo Nacional de Ciencias Naturales, Madrid, Spain; 4 Museum für Naturkunde, Leibniz-Institut für Evolutions und Biodiversitätsforschung an der Humboldt-Universität zu Berlin, Berlin, Germany; 5 Department of Medical Parasitology, Medical University of Vienna, Vienna, Austria; 6 Naturhistorisches Museum Wien, Universität Wien, Wien, Austria; 7 Department of Biology and Biotechnology “C. Darwin", Sapienza University of Rome, Rome, Italy; 8 Renkum, The Netherlands; 9 WorldFish Center, Natural Resource Management, Laguna, Philippines; 10 Dipartimento di Scienze dell’Uomo, dell’Ambiente e della Natura, Università di Urbino ‘Carlo Bo’, Urbino, Italy; 11 Hoogezand, Netherlands; 12 Dipartimento di Scienze Ecologiche e Biologiche, Università della Tuscia, Viterbo, Italy; 13 Museum and Institute of Zoology, Polish Academy of Sciences, Warszawa, Poland; 14 Department of Zoology, The Natural History Museum, London, United Kingdom; 15 Naturhistorisches Museum, Basel, Switzerland; 16 Dépt Systématique et Evolution, Muséum National d’Histoire Naturelle, Paris, France; 17 Natural History Museum of Denmark, University of Copenhagen, Copenhagen, Denmark; 18 Universita degli Studi della Tuscia, Dipartimento di Scienze Ambientali, Viterbo, Italy; 19 Service du Patrimoine Naturel, Muséum National d’Histoire Naturelle, Paris, France; 20 Laboratori de Parasitologia, Universitat de Barcelona, Barcelona, Spain; 21 Dept. Histoire de la Terre, Muséum National d’Histoire Naturelle, Paris, France; 22 Department of Terrestrial Zoology, Western Australian Museum, Welshpool, Western Australia, Australia; 23 Institut für Zoologie, University of Erlangen-Nürenberg, Erlangen, Germany; 24 Nationaal Natuurhistorisch Museum Naturalis, European Invertebrate Survey, Leiden, The Netherlands; 25 Zoological Museum, University of Amsterdam, Amsterdam, The Netherlands; 26 Department of Animal Taxonomy and Ecology, A. Mickiewicz University, Poznan, Poland; 27 Echternach, Luxembourg; 28 . British Antarctic Survey, Cambridge, United Kingdom; 29 Instituto de Investigacao Cientifica Tropical, Centro de Zoologia, Lisboa, Portugal; 30 Naturhistorisches Museum im Thüringer Landesmuseum, Rudolstadt, Germany; 31 Department of Biology, University of Padova, Padova, Italy; 32 Departamento de Biodiversidad y Gestión Ambiental, Universidad de Leon, Leon, Spain; 33 Flemish Entomological Society, Leefdaal, Belgium; 34 Dipartimento di protezione dei sistemi agroalimentare e urbano e valorizzazione delle biodiversità, Universita degli Studi di Milano, Milano, Italy; 35 NCB Naturalis, Amsterdam, The Netherlands; 36 Dipartimento di Scienze Ambientali, Sezione di Sistematica ed Ecologia animale e vegetale, Universita di Siena, Siena, Italy; 37 Freshwater Laboratory, Royal Belgian Institute for Natural Sciences, Brussels, Belgium; 38 Institute of Agricultural and Environmental Sciences, Estonian University of Life Sciences, Tartumaa, Estonia; University of Veterinary Medicine Hanover, Germany

## Abstract

The number of described species on the planet is about 1.9 million, with ca. 17,000 new species described annually, mostly from the tropics. However, taxonomy is usually described as a science in crisis, lacking manpower and funding, a politically acknowledged problem known as the Taxonomic Impediment. Using data from the *Fauna Europaea* database and the *Zoological Record*, we show that contrary to general belief, developed and heavily-studied parts of the world are important reservoirs of unknown species. In Europe, new species of multicellular terrestrial and freshwater animals are being discovered and named at an unprecedented rate: since the 1950s, more than 770 new species are on average described each year from Europe, which add to the 125,000 terrestrial and freshwater multicellular species already known in this region. There is no sign of having reached a plateau that would allow for the assessment of the magnitude of European biodiversity. More remarkably, over 60% of these new species are described by non-professional taxonomists. Amateurs are recognized as an essential part of the workforce in ecology and astronomy, but the magnitude of non-professional taxonomist contributions to alpha-taxonomy has not been fully realized until now. Our results stress the importance of developing a system that better supports and guides this formidable workforce, as we seek to overcome the Taxonomic Impediment and speed up the process of describing the planetary biodiversity before it is too late.

## Introduction

The number of described species on Earth is now about 1.9 million [1], with between 16,000 and 18,000 new species described every year [2]. The frontiers of biodiversity exploration and discovery are generally considered to be in the tropics [3], [4] and if the actual number of species on the planet is 5–30 million [4], at the current rate several centuries will be necessary to describe and name them all. The insufficient availability of taxonomic expertise and the gaps of knowledge in our taxonomic system represent a politically acknowledged problem, known as the Taxonomic Impediment [5]. One of the side effects of the Taxonomic Impediment, already noticed by several authors [6], [7], [8], is the strong imbalance between developed, biodiversity-poor countries and developing, biodiversity-rich countries. Another characteristic of taxonomy is that it is one of the rare scientific disciplines where non-professionals are known to play a role [9], [10], [11]. However, this role is underestimated outside the taxonomic community [12], [13], contrary to ecology [14] and astronomy [12], where amateurs are widely recognized as an essential part of the workforce.

Europe is one of the better known parts of the world in terms of biodiversity. As a testimony of this knowledge, the release of the *Fauna Europaea* database in 2004 was a landmark for European taxonomy, encapsulating the efforts of more than 450 taxonomists, coordinated by the University of Amsterdam, the University of Copenhagen and the National Museum of Natural History in Paris [15]. For the first time a comprehensive checklist was created that provided baseline reference to all the valid species of multicellular terrestrial and freshwater animals occurring in geographical Europe.

In this context, the aim of our study was to measure the growth of the taxonomic inventory of Europe, and to assess the respective weight of professional and non-professional taxonomists in the completion of the inventory.

## Results and Discussion

**Figure 1:**
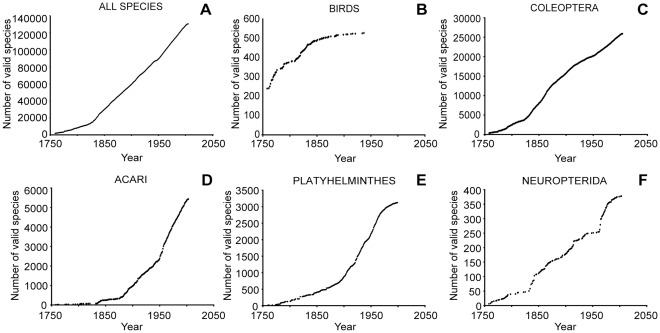
Growth of the European taxonomic inventory. Cumulative number of valid species of European terrestrial and freshwater multicellular species since Linnaeus. A: All species. B: Birds, a virtually completely inventoried compartment of European biodiversity. C: Coleoptera, where the number of valid species has steadily increased and shows no sign of levelling. D: Acari, which remained neglected for two centuries, and are now exhibiting a high discovery rate. E: Platyhelminthes, where the impression of a saturated inventory could be due to a current lack of taxonomic workforce. F: Neuropterida orders, for which the rate of description is erratic and reflects bursts of activity by a handful of taxonomists.

At the time of its first release, *Fauna Europaea* recognized 125,854 species, starting from the publication of Linnaeus’ *Systema Naturae* in 1758. Analysis of discovery rates through time showed that, despite the most formidable geographical concentration of taxonomic expertise over 250 years, a plateau had still not been reached in Europe. Three historical segments were recognized in the discovery curve of the European biota. The slope of each segment differed and most remarkably, each was significantly steeper than the previous one (Table 1 and Fig. 1a): today in Europe, more species are described each year than one century ago, and over four times more than two centuries ago. The lack of saturation in the cumulative curve indicated not only that the inventory of the European fauna was far from complete, but also that the data did not even permit an estimate of the total number of species [16]. The regular increase in the number of described species in Europe is the result of two antagonistic processes. On one hand, as more species become known it is more difficult to discover new ones, but on the other, collecting and, especially, discrimination techniques and tools are becoming more powerful, efficient and widely available, opening new avenues of species discovery in supposedly well-known faunas. When different animal groups were considered separately, discovery patterns varied (Fig. S1). Not unexpectedly, for some taxa it is increasingly rare to find new species, even with new discrimination techniques. In birds (Fig. 1b) and a few other groups such as dragonflies, saturation was reached several decades ago and the number of known species in the European fauna remains stable, except for isolated new discoveries. In other taxa, e.g. beetles (Fig. 1c), other holometabolous insects, insects as a whole, but also, perhaps more unexpectedly, freshwater fishes, the number of described species has been steadily increasing for more than 100 years. Still other groups, e.g. mites (Fig. 1d), nematodes and springtails are experiencing a modern explosion of species descriptions after a stasis that lasted until the first half of the 20th century. Rates of descriptions are decreasing for groups such as free-living flatworms (Fig. 1e) and thrips, suggesting that we are getting closer and closer to knowing them all. However, to some extent this could reflect the drying up of taxonomic expertise: if there is no specialist to recognize new species, we may gain the impression of a saturated inventory [17]. Indeed, a few groups show temporary plateaus, but these reflect the temporal variation in availability of a relevant taxonomic workforce [8] rather than the saturation of species discovery, as for instance for annelids from the 1930s to the 1950s (Fig. S1) and neuropterid insects from the 1930s to the 1960s (Fig. 1f).

**Table 1:** Growth in European taxonomic inventory summary.

Period	Estimates (n.sp.year^−1^)	95% CI
1758−1821	177.2	170.6−183.8
1822−1954	606.5	604.3−608.7
1955−2004	778.3	768.7−787.9

Results of the segmented model fitted to the *Fauna Europaea* dataset: for each historical segment, estimates of the number of new species described per year and 95% confidence interval.

**Figure 2:**
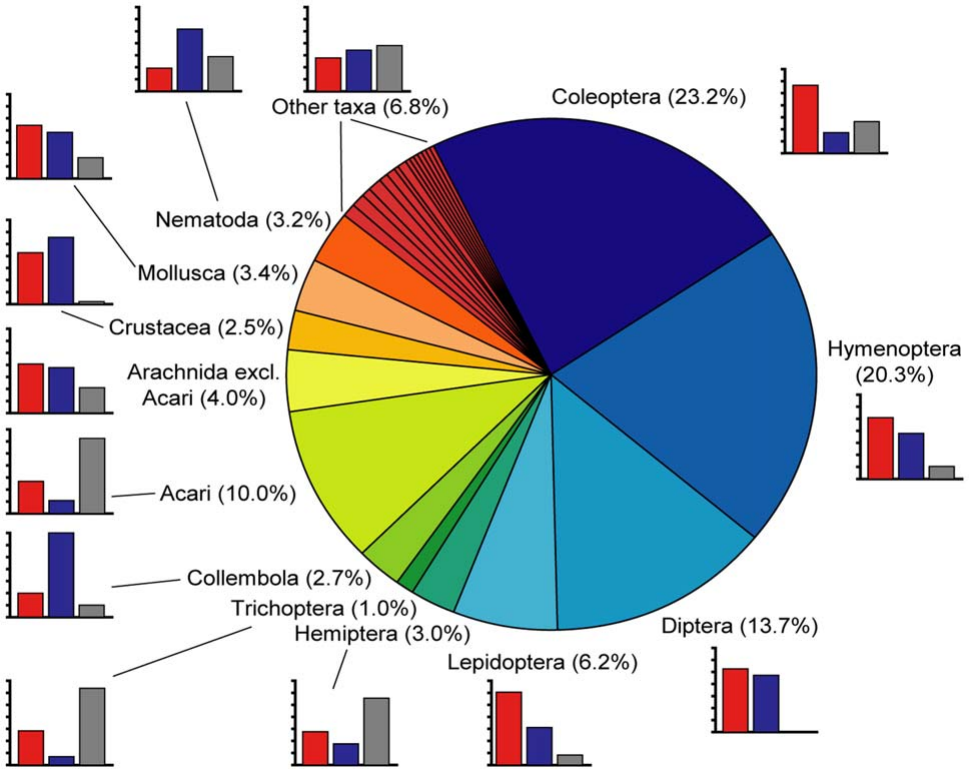
Current descriptions of new species in Europe. New species described from Europe in 1998–2007, expressed as percentages of the total of 5,881 species. Taxa representing less than 1% of the total are grouped. For each taxon, the percentages described by non-professional taxonomists (red), professional taxonomists (blue) and taxonomists whose status was unknown (grey) are indicated in histograms. Y-axis range on all histograms is 0–70%.

As *Fauna Europaea* only recorded taxa up to 2004, current taxonomic activity in Europe was assessed with data extracted from the *Zoological Record* with reference to terrestrial and freshwater species. This extraction showed that between 1998 and 2007, 5,881 new species were described from European countries, i.e. an average of 643.8 new species per year. This figure was lower than the figure obtained from the *Fauna Europaea* database (778.3 n.sp.year^−1^ since 1955 [18]). This discrepancy was expected since the *Zoological Record* coverage is considered to be incomplete, covering ca. 90% of published names, the remaining being published in sources incompletely scanned by the *Zoological Record* [19]. *Fauna Europaea* coverage is more comprehensive because it is delivered by taxonomic specialists and includes species which were described from countries outside Europe, such as from North Africa and North America, and subsequently discovered in Europe. The taxonomic composition of species described in 1998–2007 is shown in Fig. 2: 4,287 (72.9%) of the new species were Hexapoda, and the mega-diverse insect orders Diptera, Lepidoptera, Hymenoptera and Coleoptera taken together represented 63.3% of all the new species. After insects, arachnids were the second major taxon in terms of contributing numbers of new species in Europe. However, while newly named species represent the most visible part of taxonomy, revisions constitute another necessary aspect of this discipline as they allow taxa to be better characterized and reduce the number of unwarranted nominal species arising from taxonomic inflation [20]. Quite frequently, revisions lead to synonymization of nominal species, sometimes long after their original description [21]. In the European fauna, during the same time span (1998–2007), 1,998 species had been placed in synonymy, i.e. the net increase of the species inventory was 4,093 species.

We defined two groups of taxonomists, according to their professional status (see details in Material and Methods):

- Professional taxonomists: people paid to do taxonomy, either having a formal position in taxonomy, or students;
- Non-professional taxonomists: people who do not hold a position in which they are remunerated for performing taxonomy, i.e. amateurs in the broad sense and retired professionals.

This professional status was assessed for 1,000 unique authors (out of 1,323) who described new species in 1998–2007: 42.8% were professional taxonomists, (taxonomists with formal taxonomic positions: 41.2%; students: 1.6%). The others were non-professional: retired professional taxonomists (10.5%) and amateurs in the broad sense (46.7%). However, professional taxonomists described only 37.8% of the new species in the study period, i.e. non-professional taxonomists described proportionally more than those in formal taxonomic positions. Contrary to a common belief outside the taxonomic community, non-professional taxonomists do not focus only on charismatic groups: during the study period, they have described 52.7% of the new Diptera species and 26.7% of the new mite species (Fig. 2). Similarly, these non-professionals are fully involved in revisionary work: among the 1,186 species placed in synonymy in the study period and for which the professional status of the synonymising author was known, 46% were placed in synonymy by paid professional taxonomists.

Results similar to those based on European terrestrial and freshwater multicellular animals may not be obtained for other branches of the tree of life or in other parts of the world. For instance, unicellular species are today probably only described by taxonomists having access to sophisticated laboratory equipment, therefore eliminating non-professional taxonomists. However, this has not always been the case, as shown by the case of Alfred Kahl, a non-professional ciliatologist who described 17 new ciliate families, 57 new genera and ca. 700 new species in the first half of the 20th century [22]. Similarly, deep-sea species can only be collected by academic teams using expensive boats or submersibles. But even in this case, non-professional taxonomists have their share in the description of collected marine species, as is known for instance for mollusks [10]. However, we do not know the importance of their contribution, for instance for marine crustaceans, nematodes or fish. Similarly, the weight of amateurs is probably more important in Europe, North America and Australia than in emerging countries which are very active in taxonomy, such as China or Brazil: this should be tested when data are available. For plants, non-professionals play a role in species description, especially in charismatic groups such as orchids and cacti which focus the attention of many garden clubs and collectors. However, no published data seem to be available to measure their contribution.

Bearing in mind that our results indicate that it is not yet possible to reliably quantify the number of undiscovered species in Europe [18], taxonomy as a scientific discipline should be strengthened if we truly intend to document biodiversity and produce the tools needed for its conservation and sustainable use. Even in Linnaeus’ own continent, narrow-range endemics and other rare species, such as habitat specialists, remain only partially documented, despite the obvious consequences in terms of conservation [23]. Legal restrictions on specimen sampling [24], inadequate funding [25] and poor recognition of taxonomic work through bibliometry [26] are known to hamper taxonomy. This discipline is in crisis where institutional support is concerned: funding is lacking for recruitment and training, and taxonomy positions in academic institutions are being replaced by positions dealing with ecology or conservation, and taxonomy in general loses weight in curricula. Taxonomy is usually downgraded by decision-makers, who think that the inventory of European biodiversity was completed at the end of the 19th century. However, paradoxically, taxonomy as a science is more dynamic than ever for several reasons: interest in and access to new ecological niches (e.g. deep caves, river underflows, interstitial layer between rock and soil, high-altitude ice-cracked rocks, glacial cryoconite holes or anchialine caves); increasing numbers of active taxonomists [27]; use of new techniques (molecular techniques of course, but other techniques as well such as sonograms for insects or bats, x-ray microtomography of amber fossils and arthropod skeleto-muscular anatomy).

However, several factors are limiting productivity in taxonomy. The first, raised during *Fauna Europaea* regional and thematic validation workshops, is the absence of an effective policy-supported business plan to achieve a complete inventory of biodiversity at the European level, and even at a national level (with the exception of Sweden and its *Swedish Taxonomy Initiative* and Spain with *Fauna Ibérica*). There are numerous global, regional and national initiatives to collate existing knowledge into taxonomic databases and provide access to this knowledge through web-based portals (e.g. Catalogue of Life, the Pan-European Species Directories Infrastructure, the World Register of Marine Species and the Freshwater Animal Diversity Assessment, to name a few). In contrast, initiatives such as the Census of Marine Life, that set themselves the task of exploring the unknown and undocumented, are much less numerous and less well-supported [28]. In Europe, the 5th to 7th Framework Programs for Research and Technological Development have provided important funding to structure and manage taxonomic information at the European level, with projects such as *Fauna Europaea* and the *European Register of Marine Species*, but there is no coordinated European funding to explore biodiversity and produce taxonomic information. For European decision-makers, European biodiversity appears to be fully known and the main remaining task is to organize existing data. The situation is different in the United States, where the NSF program Planetary Biodiversity Inventories aim at funding “research and collecting activities that are designed to discover and document the biological species diversity of all forms of life on Earth". Similarly, the Australian Biological Resources Study program funds the production of knowledge on the biota of Australia. European funded programs to explore and document specific taxa or limited areas exist and are appreciated, but there is an urgent need for a comprehensive strategy for all of Europe, which should preferably target speciose and non-charismatic groups such as mites, rove beetles and nematodes, not to mention protists [29].

A second limiting factor preventing the completion of an inventory of European biodiversity is the non-availability of taxonomists, just as in the tropics. Professional taxonomists are not numerous enough, and they cannot spend all their time on species descriptions, as they also have to deal with administrative tasks, fund-raising and teaching. The attrition of the taxonomic workforce has been mourned in numerous position papers and blueprints [9], [30], but rather than lament this state of affairs, we suggest that more attention should be given to ways of enhancing the scientific production of non-professional experts. Increasing access to cyber-infrastructures such as digitized literature, images of type specimens and nomenclatural databases benefits all taxonomists, regardless of their status: non-professionals have access to a wealth of taxonomic information. But to enhance their efficiency, it is also important to find ways of making literature of limited distribution more accessible online, to encourage them to publish their results in peer-reviewed journals, and to prompt deposition of types in public institutions. We also believe that All Taxa Biodiversity Inventories [31] and other large-scale inventory programs with defined and coordinated objectives can provide a framework within which non-professionals should be integrated. Several other recommendations have been made [11], e.g. facilitating specimen loans from academic collections, helping with collecting and research permits or providing educational opportunities on new procedures and techniques.

Rising molecular techniques (e.g. barcoding), which are becoming an essential component of high-standard systematics, are another important issue in this context. They are largely outside the scope of the non-professional experts due to expense. Moreover, despite the democratization of sequencing, it is not a panacea, as interpretation of sequences is non trivial. Being able to interpret sequences implies academic training, which some of the non-professionals do not have. But expertise of highly proficient non-professional experts is needed to put names on specimens which are tested with molecular techniques. These experts should not be seen as second-class taxonomists: skills needed to generate high-standard morphological data are no more trivial than those needed to correctly interpret molecular data, as they imply testing a whole series of detailed hypotheses beginning with homology concepts and following through to hypotheses of synapomorphy, and understanding the abilities and limitations of the various theoretical criteria by which homology and homoplasy may be teased apart. This given, the challenge is to avoid a splitting of taxonomy in two parts: taxonomy based on molecular approaches only on one side, producing new concepts and hypotheses (i.e. phylogenetic trees), but which does not necessary lead to new species descriptions, and more traditional taxonomy, based solely on morphology, which is the only one accessible to amateurs in the traditional sense. Integrative taxonomy, including several tools ranging from morphology to molecular techniques, is unrealistic in many cases; therefore, we need both approaches, integrative and traditional, at an equally high standard, i.e. performed by educated and trained taxonomists.

Networks linking paid professionals and non-professional taxonomists could be developed to facilitate more efficient combination of molecular methodologies with alpha taxonomy. Such enhanced cooperation between molecular-oriented professionals and morphology-oriented taxonomists could be organized through small grants (e.g. the *SynTax* grants jointly administered by the Linnean Society and Systematics Association), or by systematically incorporating taxonomic specialists (professionals or non-professionals) within larger more encompassing grants. This type of cooperation is already seen in astronomy, where access to high standard technology for amateurs has triggered a new era in the collaboration with professionals [12]. Non-professional astronomers who now use state of the art equipment are organized in networks, hold research grants and take part in large-scale projects in collaboration with professionals, a situation which has opened fruitful fields of research and should be transposed into taxonomy. An example of these collaborative efforts is found with the program *Sphingidae Barcode of Life*, where 87% of the 1,470 world hawkmoth species have already been barcoded. The barcoding is performed at the Biodiversity Institute of Ontario (University of Guelph, Canada), but the specimens are provided and identified by a team of taxonomists including several non-professional experts. One of the two co-chairs of the program is an amateur, and only two out of the ten associated “sphingid expert taxonomists" have an academic position related to taxonomy [32].

However, good, traditional taxonomy should not be sold for a partnership in molecular studies. The most essential contribution of non-professionals is their broad, deep, long-term experience with their group, which is more than just a plausibility-control for the molecular analyses based on putting names on specimens. For instance, rare species are a widespread characteristic of biodiversity, and biodiversity surveys yield an important proportion of singletons (species represented by only one specimen) and uniques (species collected on only one locality) [33], [34]. Despite the fact that the concept of rarity has not been integrated by molecular systematic techniques, it has been shown that as much as 17% of new species described are based on singletons [35]. This practice has been criticized [36], but it is nevertheless unavoidable if we aim at describing rare species. This situation leaves room for traditional, morphology-oriented taxonomists, often non-professionals, who describe species that cannot be dealt with by molecular techniques.

Doing taxonomy implies being able to analyze and interpret complex data on a wide range of subjects, from morphology to molecular sequences, in an accurate, explicit, and testable way with sophisticated tools. In particular, collecting comparative morphology data is not trivial, and needs as much specific training as molecular taxonomy. However, professional status is not necessarily linked with the level of taxonomic skills needed to do comparative morphology and to produce sound revisionary studies: many non-professional taxonomists have a relevant PhD and continue doing taxonomy during their spare time while holding a position in a different branch of activities, e.g. biomedical industry, informatics, ecology or editorial work. With proper training, working on a voluntary basis does not imply second-class taxonomy. If the largest part of the work of non-professionals concerns alpha taxonomy, which is a precondition for comparative morphology, these non-professional experts can reach a high standard of excellence, and may produce information about biological complexity (anatomy, ecology, behaviour, phenology) of great evolutionary and environmental interest and which does not depend upon using molecular techniques. Involving non-professionals does not mean that taxonomy will deliver lower quality results to increase its productivity, but rather that the professional taxonomist community ensures that the non-professional colleagues are properly trained when needed.

The results presented in this paper are common knowledge of the taxonomic community, but have rarely, if ever, been quantitatively assessed. Moreover, they have been completely ignored outside the taxonomic community. In particular, decision-makers and experts in commissions, panels and boards where funding is allocated to research fields, who are rarely taxonomists, may not realize that European biodiversity is by no means a completed task but rather a frontier of exploration. This probably accounts for the fact that most European countries and organizations allocate more funds to organize and analyze already existing taxonomic information than to biodiversity exploration. It is thus important that our results reach a large audience outside the taxonomic community. However, the importance of the non-professional workforce could be poorly interpreted. Among tenured professionals, its weight can be underestimated, as they consider it as a danger for the discipline: a large non-professional workforce, working for free, could carry the wrong message to decision-makers that taxonomy does not need funding. This danger should not be underestimated, and taxonomists should advocate the better integration of non-professionals in their community. Non-professional taxonomists, who cannot resist doing descriptive work on their favorite group during their free time – they simply love it -, will always be there, with or without incentive from the professionals. It is an opportunity to strengthen this discipline, and efforts should be made to take advantage of this situation.

## Materials and Methods

We refer to Europe as a geographical entity, extending from the Ural Mountains to the Macaronesian islands, as defined for *Fauna Europaea* [15].

The *Fauna Europaea* database as of January 2005 was used to measure the growth of taxonomic discovery in Europe from 1758 to 2004. Only valid species were considered. Dates of publication of species names were used to calculate cumulative numbers of valid species, in order to show the increase due to genuine species discoveries and not to changes in species concept.

The analysis of current trends (1998–2007) is based on a dataset extracted from the *Zoological Record*, with the keywords “sp. nov.", “syn. nov." and European country names. From this dataset, we excluded marine species, unicellular organisms, fossils, and taxa from the Asiatic parts of Russia and Turkey, as being outside the remit of this paper. Taxa described in 2007 were not included in the average number of new species per year, because they were still incompletely captured in the *Zoological Record* when the research was performed in 2009.

The professional status of authors having described European species in 1998–2007 was assessed by *Fauna Europaea* Group Coordinators. The relevant Group Coordinator was asked to assign the first authors to one of four categories: professional taxonomists (a scientist who gets a salary primarily for taxonomic work), students, retired professional taxonomists, volunteer taxonomist (i.e. unpaid taxonomist, getting his/her income from any other source, academic or not, i.e. *amateur* in conventional terminology – this does not carry any judgment on the quality of the work done). We then classified taxonomists based on whether or not they get their income from doing taxonomy. This income-based categorization gives two types of taxonomists:

- Professional taxonomists, who are paid to do taxonomy: those having a formal taxonomic position in a research facility for instance, and students who benefit from grants to become professional taxonomists. An academic researcher dividing his or her working time between research on conservation biology (or other non-taxonomic biological discipline) and taxonomy would be categorized as a professional taxonomist, as long as taxonomy is not incidental in his or her official position;
- Non-professional taxonomists, who do taxonomy on a volunteer basis: this include amateurs in the broad sense, i.e. people who do taxonomy for pleasure, during their spare time, and get their income from other occupations. Among these amateurs are people who followed curricula in taxonomy but did not get a position in this discipline. Retired taxonomists, who are often very active, are also included in this category because they do not rely on doing taxonomy to get their income.

We acknowledge the fact that the status of non-financed Master/PhD candidates is in-between, as they are not paid but are nevertheless assigned to the “professional taxonomists" category. However, students (financed and non-financed) represent 1.6% of authors only, and trends and conclusions would not be significantly affected by an attribution of non-financed students to one or the other category.

If the author was not known by the relevant Group Coordinator, and if no conclusive information could be found from other sources such as personal webpages or addresses given in recent publications, the author was discarded from the analysis. As a result, out of 1,323 different authors, the status of 323 could not be clarified.

Testing of the variation in species description rates was carried out in two steps. First, the existence of breaking points in the relationships between cumulative numbers of species and year of description was tested with Davies’ test [37], and secondly a segmented model [38] was fitted to the dataset using R Package *segmented* version 0.2–7.

## Supporting Information

Figure S1 **Cumulative number of valid species of terrestrial and freshwater multicellular animals recorded in Europe.** Numbers of valid species described since Linnaeus (1758) are plotted against the description year for selected phyla, major classes and major insect orders. These groups are not of equivalent taxonomic rank but were divided as such to demonstrate occasional opposing trends within representative taxa.
(TIF)
