# Supplementary figures and images for: New Species in the Old World: Europe as a Frontier in Biodiversity Exploration, a Test Bed for 21st Century Taxonomy
Source: PLoS One. 2012 May 23;7(5):e36881. doi: 10.1371/journal.pone.0036881 (PMC3359328; doi:10.1371/journal.pone.0036881)

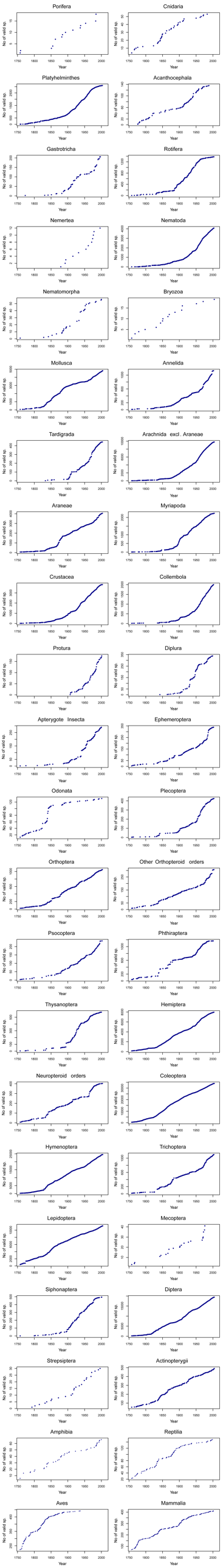

Supplement: Figure S1 — Cumulative number of valid species of terrestrial and freshwater multicellular animals recorded in Europe. Numbers of valid species described since Linnaeus (1758) are plotted against the description year for selected phyla, major classes and major insect orders. These groups are not of equivalent taxonomic rank but were divided as such to demonstrate occasional opposing trends within representative taxa. (TIF) [file pone.0036881.s001.tif]
